# Pornography consumption and psychosomatic and depressive symptoms among Swedish adolescents: a longitudinal study

**DOI:** 10.1080/03009734.2018.1534907

**Published:** 2018-11-09

**Authors:** Magdalena Mattebo, Tanja Tydén, Elisabet Häggström-Nordin, Kent W Nilsson, Margareta Larsson

**Affiliations:** a Department of Women’s and Children’s Health, Uppsala University, Uppsala, Sweden;; b School of Health, Care and Social Welfare, Mälardalen University, Västerås, Sweden;; c Center of Clinical Science, Uppsala University, Västmanland County Hospital, Västerås, Sweden

**Keywords:** Adolescents, longitudinal, pornography, psychological health

## Abstract

**Background:** The aims of this longitudinal study were to identify predictors for continued pornography consumption and to investigate pornography consumption in relation to psychosomatic and depressive symptoms among a group of adolescents in Sweden.

**Methods and materials:** A longitudinal study in classroom environment in 53 randomly selected senior high school classes in mid-Sweden in years 2011 and 2013. Out of 477 participating boys and 400 girls in 2011, 224 boys (47%) and 238 girls (60%) participated in 2013.

**Results:** Higher pornography consumption at baseline and being born outside Sweden predicted continued pornography consumption at follow-up (adjusted *R^2^* = 0.689).

Psychosomatic symptoms at follow-up were predicted by higher pornography consumption at baseline (adjusted *R^2^* = 0.254), being a girl, living with separated parents, and attending a vocational high school program. By contrast, depressive symptoms at follow-up were predicted by less pornography consumption at baseline (adjusted *R^2^* = 0.122) and being a girl.

**Conclusions:** Pornography consumption may, for some individuals, be associated to mental health issues. Differences between teenage boys and girls and between adolescents with diverse ethnic backgrounds imply that counseling and discussion about pornography need to be adjusted and individualized.

## Introduction

Strong links between pornography consumption and sexual perceptions and behaviors have previously been demonstrated (1–5), but the reported associations between pornography consumption and mental and physical health indicators are not as strongly linked (1–3) and the state of knowledge remains incomplete.

The average age of first Internet exposure to pornography has been reported to be 11 years, and the largest consumer group was found among 12–17-year-olds (2). Policies to minimize the long-term negative social impact of such use have been proposed (2). Pornography consumption among adolescents has increased (6–8), and frequent use of pornography among boys is associated with sexual experiences, obesity, peer relationship problems, high alcohol consumption, and distress (6,9,10). Pornography consumption may have detrimental effects on people other than the consumer, e.g. creating a hostile environment at school (if consumed at school), sexual harassment, and sexual aggression behaviors. Negative gender attitudes among boys overlap with regular use of online pornography, and it is argued that pornography is both underpinned by and perpetuates gender inequality (11). At the same time, there has been a significant increase in the incidence of mental health problems among individuals and couples, some of whom attest to the negative influence of pornography consumption on their lives (12). In Sweden, almost all teenage boys and more than 50% of teenage girls have consumed pornography (6,7,10).

Pornography and sexually explicit content can be found in all types of media and may alter the user’s mental, emotional, and social traits (13–17). In a study of adults, depressive symptoms were associated with the use of sexually explicit material online, both among men and women (1). In the same study, consumers of such media had almost six times higher odds for diminished mental health, over eight times higher odds for lower health status, and over five times higher odds for poorer quality of life compared with controls (1). Twice as many 10–17-year-old online seekers of pornography reported clinical features of major depression compared with their peers (18).

Despite this, the specific nature and directionality of the association between depressive symptoms and the use of sexually explicit media remain uncertain (1,13–17). Other studies have reported pornography as a way to deal with feelings of discomfort or emotional stress by male adults (1,13) and by children (19). Psychosomatic symptoms among adolescents such as headache, abdominal pain, backache, irritability, nervousness (20), and depressive symptoms (21) are rising in the Western world (20,21). In a Swedish study, 6% of boys and 20% of girls reported several psychosomatic symptoms, and 16% of teenage boys and 32% of teenage girls fulfilled the criteria for depression (22). Whether this high prevalence is associated with the increased use of pornography remains uncertain. Associations between sociodemographic factors such as living with one parent, low parental education, low social capital, and low general social trust and psychosomatic and depressive symptoms have been described (21–23). However, knowledge about associations between pornography consumption over time and sociodemographic factors such as high school program, living situation, family situation, or ethnic background is scarce. To develop and implement sexual health intervention programs, it is important to investigate whether frequent users of pornography over time differ from other users or non-users in terms of sociodemographic predictors and mental health.

The aims of this longitudinal study were to identify sociodemographic predictors such as ethnic background, family situation, dwelling, parents’ occupational status, and high school program in relation to continued pornography consumption and to investigate pornography consumption in relation to psychosomatic and depressive symptoms. We hypothesized that: (1) poorer sociodemographic conditions were associated to pornography consumption over time, and that (2) higher pornography consumption over time would be related to psychosomatic and depressive symptoms.

## Materials and methods

This longitudinal classroom survey took place in 13 senior high schools in Fagersta and Västerås in central Sweden. In Sweden school is compulsory from age 7 to 15 (grades 1–9). Almost all adolescents continue to senior high school, age 16–19 (high school grades 1–3). Initial data collection occurred in March through May 2011 (6), and the follow-up took place in January through March 2013. The total population of students in the two cities during the initial data collection was 2562 (1254 boys and 1308 girls). The data collected in 2011 (baseline) have previously been described (6). Data for the follow-up study were collected in 2013 (the follow-up study) from 18-year-olds, who were in their third year of senior high school, from the same classes and schools participating in 2011. The total population of third-year students in both towns in 2013 was 946 students (510 girls and 436 boys). By the second data collection, 703 (74%) of all available third-year students in the selected classes were present (339 girls and 364 boys). All students were asked to participate regardless of their participation in the first data collection to enable both cross-sectional and longitudinal analyses. Two girls and one boy declined participation. In the longitudinal analyses, 224 boys and 238 girls who completed both questionnaires participated; their mean age was 18.25 years (range 17–21, SD 0.537). These numbers represented 47% and 60% of the original sample; 253 boys and 162 girls were lost between baseline and follow-up.

A power analysis was calculated based on previously reported psychosomatic symptoms, as defined by the World Health Organization, among adolescents in the county where data collection was performed (6). The power analysis resulted in a sample of 53 randomly selected classes in 13 senior high schools with a total of 1134 students (613 boys and 521 girls). This sample size was determined to be sufficient to detect differences in psychosomatic symptoms between frequent and infrequent users of pornography, between boys and girls, and between students attending university preparatory and vocational high school programs, with a *P* value of 0.05 and a power of 80%.

The directors and principals of all participating schools granted permission for the study, and appointments were booked with the teachers. To avoid deliberate absenteeism, the students were not informed about the study before data collection. They received oral and written information about the study, and voluntary participation was emphasized. Those who did not want to participate could either leave the classroom or stay and simply read or pretend to fill in the questionnaire. The participants were asked to put their name on the questionnaire to enable comparison between the two data collection periods. This was voluntary: 7% chose to remain anonymous at the initial data collection and 3% at the follow-up. Because the voluntariness of the study was emphasized and it was possible to decline participation, it was assumed that all participants gave their informed consent by participating and no further written consent was obtained. The researcher distributed the questionnaire printed on paper, and all students received a pen to fill out the questionnaire. The students completed the questionnaire in the classroom. The desks were separated to maintain privacy. Completing the questionnaire took 15–25 minutes, after which participants placed the questionnaire in a sealed envelope and handed it over to the research assistant. All students received candy, a lottery ticket, and condoms as a token of appreciation. The local youth clinic and all school nurses were informed of the study before the data collection took place in case any participant desired personal counseling afterwards. The questionnaires were coded, and the identification page was removed from the questionnaire and kept in a locked cupboard accessible by the researchers only.

The 2011 questionnaire comprised 64 multiple-choice questions, which have been previously described (6). The same questions about demographics, pornography consumption, and psychosomatic and depressive symptoms were used in the 2013 questionnaire to enable comparison between baseline and follow-up.

A pilot test–retest, with two weeks between measurements, of the 2011 questionnaire was performed in a similar sample (*n* = 35) in another town. Correlation tests using Cohen’s kappa (nominal data) and Spearman’s rank order correlation test (ordinal data) were used, and an acceptable degree of correlation was found between the two points of measurement (mean values of 0.812 and 0.608, respectively).

### Measures

#### Sociodemographics

Sex was coded as male (=0) or female (=1). The participants indicated the high school program in which they were enrolled (university preparatory [=0] or vocational [=1]), dwelling (rented [=0] or parent-owned [=1]), family members with whom they lived (living with two parents [=0], one parent [=1], or other [=2]), and ethnic background (born in Sweden/the Nordic Countries [=0] or born outside Sweden [=1]). The ethnic variable will be described as born in Sweden and born outside Sweden.

#### Pornography consumption

Participants reported the frequency of their pornography consumption. The response options were: never (=0), once a year or less (=1), a few times a year (=2), a few times a month (=3), weekly (=4), daily (=5), or several times daily (=6).

#### Psychosomatic symptoms

Participants reported their frequency of six psychosomatic symptoms: headache, stomachache, nervousness, irritation, stress, and trouble sleeping. Response options were never (=0), seldom (=1), sometimes (=2), often (=3), or always (=4). Since these psychosomatic symptom measures were sufficiently correlated (Cronbach’s alpha 0.75) (22) a summation index was created with a range of 6–30 points and used as a dependent variable.

#### Depressive symptoms

Symptoms of depression were estimated using an adult version of the Depression Self-Rating Scale (DSRS) based on the *Diagnostic and Statistical Manual of Mental Disorders* (DSM-IV) A and C criteria for depression. We used the DSRS with the DSM-IV A and C criteria, which have a reported sensitivity of 86% and specificity of 75% for an expert-rated diagnosis in adult psychiatric inpatients and outpatients (24). Dichotomous answers to 16 questions about depressive symptoms occurring during the past two weeks were used to sum the symptoms reported and to calculate a depression index (22), which are referred to as ‘depressive symptoms’ and were used as a dependent variable.

### Statistical analyses

The data were analyzed using the Statistical Package for the Social Sciences (version 20; IBM Corp., Armonk, NY, USA). Generalized linear models (GLM) were used to analyze the multivariable models. We analyzed both main and two-way interactions to explain the contributions of sociodemographic factors and pornography consumption at baseline in 2011 in relation to: (1) continued pornography consumption, and (2) pornography consumption in relation to psychosomatic and depressive symptoms. When analyzing interaction effects, the interaction represents the joint effect of two variables over and above any additive combination of their separate effects. This means that one main effect may have a different impact on the dependent variable when interacting with another variable, which may sometimes appear contradictory when comparing main effect size with interaction effect size. Furthermore, since the hypothesis indicates a relationship between pornography consumption at baseline and depressive symptoms at follow-up, there should also be a relationship between pornography consumption and depression at baseline.

Since we anticipated that the association of depressive symptoms at baseline should predict depressive symptoms at follow-up, and would rule out all associations of the other independent variables (such as sex, ethnic background, etc.), which also should be related to both the predictor pornography consumption, as well as the dependent variables of depressive and psychosomatic symptoms, we performed a three-step secondary analysis. In this analysis, we calculated the association of pornography consumption at baseline: first in relation to depressive symptoms at baseline, then at follow-up. Thereafter, we analyzed the main and interaction effects of pornography consumption at baseline and follow-up in relation to depressive symptoms at follow-up. Finally, we analyzed the main and interaction effects of pornography consumption at baseline and follow-up in relation to change in depressive symptoms between baseline and follow-up. The same procedure was performed for psychosomatic symptoms. However, since the depressive symptom variable was positively skewed, we used a generalized linear mixed model (GLMM) analytical approach with a Poisson regression as a complementary statistical approach to avoid scaling artifacts.

*P* < 0.05 was considered significant for main effects, and *P* < 0.10 was considered significant for interaction effects (25). Correlations using Spearman’s rho were tested between the dependent variables (Table 1).

**Table 1.** Correlations (Spearman’s rho) between dependent variables.

Item	Pornography consumption at follow-up	Psychosomatic symptoms	Depressive symptoms
Pornography consumption at follow-up/*P*^a^	Girls = 1	Girls = 0.096/ns	Girls = 0.153/0.017
	Boys = 1	Boys=–0.012/ns	Boys = 0.019/ns
Psychosomatic symptoms/*P*^a^	Girls = 0.096/ns	Girls = 1	Girls = 0.647/<0.001
	Boys = –0.012/ns	Boys = 1	Boys = 0.539/<0.001
Depressive symptoms/*P*^a^	Girls = 0.153/0.017	Girls = 0.647/<0.001	Girls = 1
	Boys = 0.019/ns	Boys = 0.539/<0.001	Boys = 1

a Significance: *P* < 0.05.

ns: non-significant.

### Ethics

The Regional Ethical Review Board in Uppsala, Sweden approved the study (Dnr 2010/369).

## Results

### Personal characteristics

Data for all personal characteristics including sociodemographic background are presented in Supplementary Table 1 (available online). Analysis of dropouts between the two data periods showed a higher percentage of boys, participants born outside Sweden, those with separated parents, and those with unemployed fathers in 2011 compared with the 2013 follow-up. The means and standard deviations (SDs) for sociodemographic variables in relation to pornography consumption, psychosomatic symptoms, and depressive symptoms at follow-up are presented in Table 2.

**Table 2.** Sociodemographic background in relation to pornography consumption at follow-up.

Items	Pornography consumption	Psychosomatic symptoms	Depressive symptoms
	Mean (SD)	Mean (SD)	Mean (SD)
Sex			
All	2.96 (1.72)	15.88 (4.18)	2.39 (2.43)
Boys	4.25 (1.31)	13.97 (3.73)	1.79 (2.04)
Girls	1.74 (1.02), *P*^a^ < 0.001	17.68 (3.77), *P*^a^ < 0.001	2.95 (2.63), *P*^a^ < 0.001
Ethnic background			
Born in Sweden (all)	2.94 (1.71)	15.90 (4.18)	2.33 (2.45)
Born outside Sweden (all)	3.29 (1.76), ns	15.46 (4.29), ns	3.21 (2.22), ns
Born in Sweden (boys)	4.31 (1.24)	13.91 (3.70)	1.70 (2.04)
Born outside Sweden (boys)	3.84 (1.77), *P*^a^ = 0.031	14.26 (3.99), ns	2.47 (1.95), ns
Born in Sweden (girls)	1.73 (1.05)	17.66 (3.78)	2.89 (2.64)
Born outside Sweden (girls)	2.11 (1.03), ns	18.00 (3.97), ns	4.78 (1.99), ns
Household			
Living with two parents (all)	3.08 (1.72)	15.52 (4.06)	2.24 (2.36)
Living with one parent (all)	2.74 (1.71), ns	16.63 (4.15), *P*^a^ = 0.009	2.64 (2.52), ns
Living with two parents (boys)	4.32 (1.28)	13.89 (3.74)	1.73 (2.03)
Living with one parent (boys)	4.12 (1.39), ns	14.29 (3.72), ns	1.95 (2.08), ns
Living with two parents (girls)	1.72 (0.94)	17.30 (3.64)	2.79 (2.57)
Living with one parent (girls)	1.72 (1.09), ns	18.36 (3.59), ns	3.15 (2.70), ns
Dwelling			
Parent-owned (all)	2.99 (1.73)	15.75 (4.15)	2.32 (2.36)
Rented (all)	2.86 (1.68), ns	16.26 (4.27), ns	2.58 (2.65), ns
Parent-owned (boys)	4.29 (1.33)	14.00 (3.74)	1.73 (2.01)
Rented (boys)	4.09 (1.26), ns	13.87 (3.73), ns	1.96 (2.14), ns
Parent-owned (girls)	1.73 (0.97)	17.44 (3.82)	2.89 (2.52)
Rented (girls)	1.77 (1.18), ns	18.38 (3.55), ns	3.13 (2.95), ns
Father’s occupation			
Working/studying (all)	2.96 (1.72)	15.76 (4.08)	2.31 (2.39)
Unemployed/on sick leave (all)	2.84 (1.72), ns	17.22 (5.07), ns	3.30 (2.71), *P*^a^ = 0.001
Working/studying (boys)	4.26 (1.30)	14.02 (3.65)	1.73 (2.01)
Unemployed/on sick leave (boys)	4.07 (1.58), ns	13.13 (4.82), ns	2.67 (2.32), ns
Working/studying (girls)	1.72 (1.00), ns	17.44 (3.77)	2.88 (2.59)
Unemployed/on sick leave (girls)	2.00 (1.27)	20.00 (2.30), *P*^a^ = 0.001	3.73 (2.91), *P*^a^ = 0.001
Mother’s occupation			
Working/studying (all)	2.96 (1.73)	15.85 (4.12)	2.38 (2.42)
Unemployed/on sick leave (all)	2.92 (1.52), ns	16.19 (4.92), ns	2.53 (2.68), ns
Working/studying (boys)	4.29 (1.31)	13.96 (3.63)	1.82 (2.04)
Unemployed/on sick leave (boys)	3.75 (1.25), ns	14.10 (4.68), ns	1.40 (2.06), ns
Working/studying (girls)	1.73 (1.02)	17.59 (3.76)	2.88 (2.62)
Unemployed/on sick leave (girls)	1.88 (1.15), ns	18.18 (3.94), ns	3.94 (2.74), ns
Study program			
University preparation (all)	3.04 (1.72)	15.66 (4.27)	2.36 (2.45)
Vocational (all)	2.83 (1.71), ns	16.18 (4.04), ns	2.43 (2.42), ns
University preparation (boys)	4.36 (1.22)	13.58 (3.52)	1.72 (1.98)
Vocational (boys)	4.08 (1.43), ns	14.54 (3.96), ns	1.89 (2.13), ns
University preparation (girls)	1.74 (0.99)	17.73 (3.94)	2.99 (2.69)
Vocational (girls)	1.75 (1.07), ns	17.61 (3.55), ns	2.90 (2.57), ns

Mean and standard deviation (SD).

a Student’s *t* test, significance: *P* < 0.05.

ns: non-significant.

### Pornography consumption

**Figure 1.**
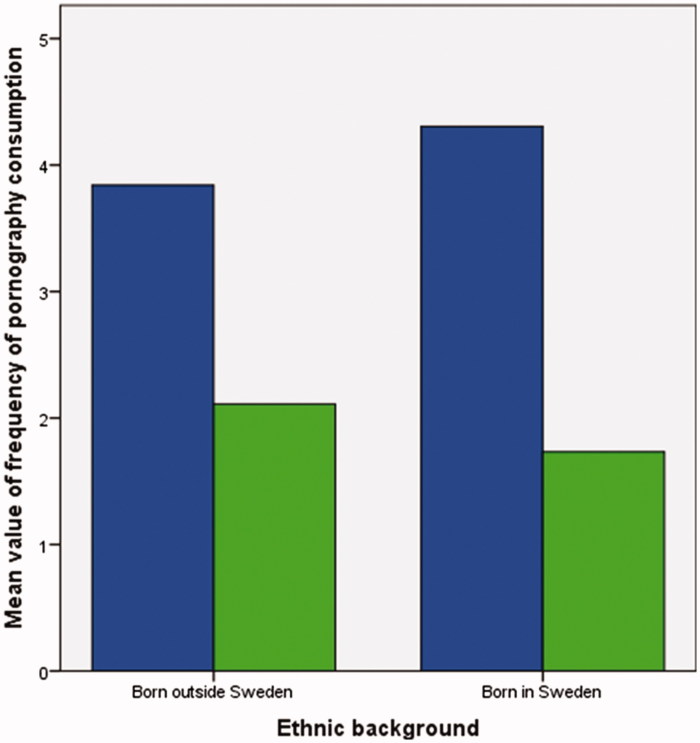
Interaction between sex and ethnic background in relation to frequency of pornography consumption. Blue bars = boys; green bars = girls. For further information about the interaction, please see Table 4.

The means and SDs for pornography consumption at baseline are presented in Table 3. There was a strong relation between use of pornography at baseline and follow-up (boys *rho* = 0.509, girls *rho* = 0.522, and total *rho* = 0.770). Pornography consumption increased between the two time points among both sexes (baseline boys *M* = 3.99, *SD* = 1.468, girls *M* = 1.61, *SD* = 1.148; and follow-up boys *M* = 4.26, *SD* = 1.309, girls *M* = 1.74, *SD* = 1.024, *P* < 0.001 respectively). The predictors of pornography consumption at follow-up were investigated. Table 4 shows the results of the multivariable GLM to identify the predictors of pornography consumption at follow-up. All variables were adjusted for significant main and interaction effects. Higher pornography consumption at baseline and being born outside Sweden were the strongest main effect predictors in this model. There were several interaction effects. The strongest interaction effect was the interaction between sex and ethnic background. This showed that girls born outside Sweden reported higher pornography consumption compared with girls born in Sweden, whereas the opposite pattern was found among boys (Figure 1). The interaction between sex and pornography consumption at baseline showed that girls were more affected by baseline consumption in relation to follow-up than boys were. However, girls with higher consumption at baseline had just marginally higher consumption at follow-up compared with boys with lower consumption at baseline.

**Table 3.** Mean value pornography consumption, depressive symptoms, and psychosomatic symptoms at baseline.

Item	Pornography consumption	Psychosomatic symptoms	Depressive symptoms
	Mean (SD)	Mean (SD)	Mean (SD)
All	2.72 (1.77)	14.89 (4.10)	2.1 (2.36)
Boys	4.03 (1.47)	13 (3.71)	1.52 (2.02)
Girls	1.49 (0.94)	16.49 (3.80)	2.65 (2.53)

Mean and standard deviation (SD).

**Table 4.** Main and interaction effects in relation to pornography consumption at follow-up (adjusted *R^2^* = 0.689).

Item	*F*	*P*^a^	Eta-square
1. Sex	4.13	0.04	0.009
2. Ethnic background	13.84	<0.001	0.030
3. Household		ns	0.000
4. Dwelling	5.13	0.02	0.011
5. High school program	5.17	0.02	0.012
6. Pornography consumption at baseline	98.81	<0.001	0.183
1 × 2	14.52	<0.001	0.032
1 × 6	4.50	0.03	0.010
2 × 4	5.27	0.02	0.012
2 × 5	3.21	0.07	0.007
2 × 6	13.98	<0.001	0.031
5 × 3	2.97	0.05	0.013
5 × 6	3.38	0.07	0.008

a Significance: *P* < 0.05 main effects, *P* < 0.1 interaction effects.

ns: non-significant.

The interaction between ethnic background and pornography consumption at baseline showed that adolescents born outside Sweden reported higher pornography consumption at follow-up compared with adolescents born in Sweden (Table 4).

### Psychosomatic and depressive symptoms

Similar GLMs were used to analyze pornography consumption in relation to psychosomatic and depressive symptoms. Predictors were analyzed separately in relation to psychosomatic symptoms and depressive symptoms at follow-up. We analyzed both main and two-way interaction effects. All variables were adjusted for significant main and interaction effects.

Table 5 shows that psychosomatic symptoms at follow-up were predicted by higher pornography consumption at baseline, being a girl, living with separated parents, and attending a vocational high school program. The strongest interaction effect was between pornography consumption at baseline and attending a vocational program. This showed that adolescents who reported higher pornography consumption at baseline attending a vocational program reported psychosomatic symptoms to a higher extent compared with peers also attending a vocational program. This was in contrast to adolescents attending a university preparatory program, where adolescents who reported higher pornography consumption at baseline reported psychosomatic symptoms to a lower extent compared with peers also attending a university preparatory program (Table 5).

**Table 5.** Main and interaction effects of psychosomatic symptoms at follow-up in relation to pornography consumption (adjusted *R^2^* = 0.254).

Item	*F*	*P*^a^	Eta-square
1. Sex	54.84	<0.001	0.110
2. Household	4.84	0.008	0.014
3. Father’s occupational status		ns	0.000
4. Mother’s occupational status		ns	0.002
5. High school program	6.24	0.01	0.014
6. Pornography consumption at baseline	5.93	0.01	0.013
7. Pornography consumption at follow-up		ns	0.005
1 × 3	7.75	0.006	0.017
2 × 4	5.01	0.007	0.022
5 × 6	10.44	0.001	0.023
6 × 7	3.48	0.06	0.008

a Significance: *P* < 0.05 main effects, *P* < 0.1 interaction effects.

ns: non-significant.

**Figure 2.**
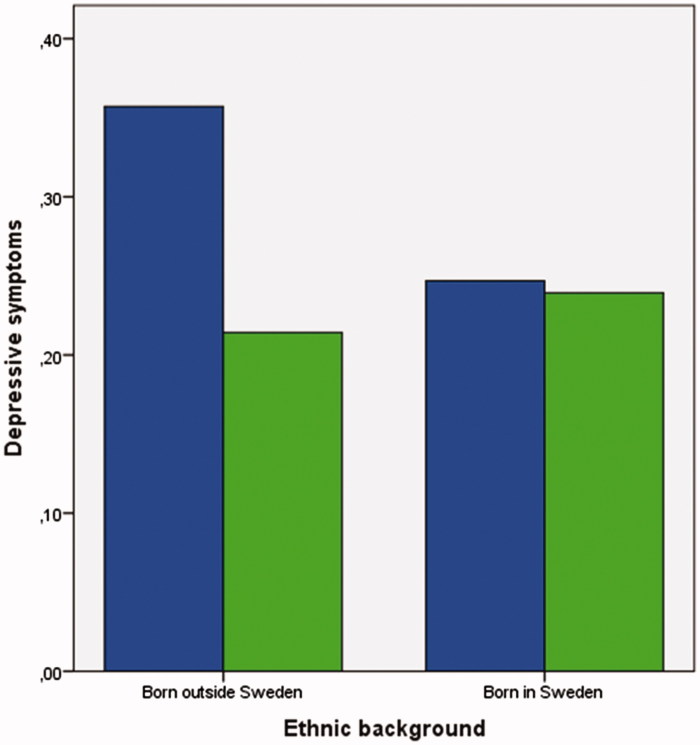
Interaction between ethnic background and pornography consumption in relation to depressive symptoms. Blue bars denote users with lower pornography consumption at follow-up, whereas green bars denote higher consumption. For further information about the interaction, please see Table 6.

Table 6 shows that in a GLM depressive symptoms at follow-up were predicted by lower pornography consumption at baseline and being a girl. However, in a Poisson regression model, the direct effect of being a girl, been born outside Sweden, living in a rental apartment, having an unemployed father, and pornography consumption at baseline were related to depressive symptoms. Furthermore, in the GLM the strongest interaction effect was between sex and pornography consumption at baseline. Girls who reported higher pornography consumption at baseline reported depressive symptoms to a higher extent, whereas the opposite pattern was found among boys (Table 6). Interestingly, all interaction effects, except sex and ethnic background, were verified in the Poisson regression model. The interaction between pornography consumption and ethnic background showed that adolescents born outside Sweden reported depressive symptoms to a higher extent if they had lower pornography consumption, whereas adolescents born outside Sweden with higher pornography consumption were those with least depressive symptoms (Figure 2).

**Table 6.** Main and interaction effects of depressive symptoms at follow-up in relation to pornography consumption (adjusted *R*^2^ = 0.122) analyzed with a general linear model and complementary statistics in a Poisson regression.

Item	*F*	*P*^a^	Eta-square	Chi-square	*P*^a^
1. Sex	7.28	0.007	0.016	27.863	<0.001
2. Ethnic background		ns	0.001	9.175	0.002
3. Household		ns	0.005	5.868	0.015
4. Father’s occupational status		ns	0.001	6.485	0.011
5. Mother’s occupational status		ns	0.002	1.246	0.264
6. Pornography consumption at baseline	12.10	0.001	0.027	12.589	0.050
1 × 2	5.33	0.02	0.012	0.143	0.706
1 × 5	5.58	0.02	0.013	4.907	0.027
1 × 6	12.46	<0.001	0.027	11.447	0.022
2 × 6	5.08	0.02	0.011	18.516	0.001
3 × 4	4.31	0.01	0.019	24.290	<0.001
3 × 5	2.85	0.05	0.013	6.786	0.009

a Significance: *P* < 0.05 main effects, *P* < 0.1 interaction effects.

ns: non-significant.

The fact that depressive symptoms at baseline was the strongest predictor for depressive symptoms at follow-up (*rho* = 0.575) and ruled out all other variables (which were related to both pornography consumption and to depression) in a multivariable regression made it difficult to adjust for depressive symptoms at baseline in the model predicting depressive symptoms at follow-up. Therefore, we further explored the relationship between pornography consumption and depressive symptoms in some additional analyses.

In the first step, pornography consumption at baseline was related to depressive symptoms at baseline (*F* = 7.735, *P* = 0.006). In the next step, pornography consumption at baseline was related to depressive symptoms at follow-up (*F* = 4.077, *P* = 0.044) as was pornography consumption at follow-up (*F* = 5.214, *P* = 0.023). Furthermore, the interaction of pornography consumption at baseline and follow-up pointed at an interaction effect (*F* = 3.235, *P* = 0.073), adjusted for sex (*F* = 32.154, *P* < 0.001) and ethnic background (*F* = 6.575, *P* = 0.011).

However, in a final analysis investigating the change in depressive symptoms between baseline and follow-up, we found that pornography consumption at baseline (*F* = 9.077, *P* = 0.003), pornography consumption at follow-up (*F* = 5.985, *P* = 0.015), as well as the interaction pornography consumption at baseline × pornography consumption at follow-up (*F* = 12.189, *P* = 0.001) predicted the change in depressive symptoms, adjusted for sex and ethnic background, which were no longer significant. This indicates that there was a relation between pornography consumption and depressive symptoms, and that pornography consumption may have an impact on the development of depressive symptoms among some groups of adolescents (data not shown).

In the secondary analysis of psychosomatic symptoms, the association between baseline and follow-up was *rho* = 0.662. Therefore the same procedure was followed for the association between pornography consumption at baseline and follow-up, in relation to psychosomatic symptoms. In short: the main effect of pornography consumption at baseline (*F* = 8.656, *P* = 0.003), as well as the interaction pornography consumption at baseline × pornography consumption at follow-up (*F* = 5.263, *P* = 0.022), predicted psychosomatic symptoms at follow-up. However, the main effect of pornography consumption at follow-up (*F* = 3.375, *P* = 0.067) was just borderline significant when adjusting for the interaction effect. In a final analysis we investigated the association between pornography consumption at baseline and follow-up, in relation to change in psychosomatic symptoms. The main effect of pornography consumption at baseline (*F* = 6.639, *P* = 0.010), as well as the interaction pornography consumption at baseline × pornography consumption at follow-up (*F* = 6.901, *P* = 0.009), predicted the change in psychosomatic symptoms at follow-up. However, when adjusting for the interaction effect, the main effect of pornography consumption at follow-up (*F* = 1.428, *P* = 0.233) was not significant (not shown in Tables).

## Discussion

Our hypotheses were partly confirmed. Some sociodemographic variables, such as sex and ethnicity, were associated to pornography consumption over time. Both psychosomatic and depressive symptoms at follow-up were predicted by pornography consumption (at baseline and at follow-up) as well as the change in psychosomatic and depressive symptoms between baseline and follow-up.

Higher pornography consumption at baseline and being born outside Sweden had strong predicting main effect size for continued pornography consumption from baseline to follow-up. The findings in the multivariable model of depression were verified in a Poisson regression model, which rules out the possibility for scaling artifacts.

The interaction effect between pornography consumption at follow-up, sex, and ethnic background showed contradictory findings, perhaps as a consequence of cultural gender norms (26,27). Girls born outside Sweden reported higher pornography consumption compared with girls born in Sweden, whereas the opposite was found among boys. The reason for this remains unclear. One possible explanation could be that ethnic background could influence ways and possibilities of expressing sexuality.

During adolescence, sexual exploration and expression are common and normal (28–30). In general there is a liberal and understanding attitude toward adolescent sexuality in Sweden. This may contrast with perceptions of individuals and families who have migrated from other countries. It is possible that adolescents born in countries outside Sweden have limited possibilities for discussing sexuality issues at home and limited access to youth-friendly services (31) and may use pornography as a source of information about sexuality. However, these results should be interpreted with some caution, as we did not ask for participants’ specific country of birth or about how many years they had lived in Sweden. We recommend further research to confirm these findings.

On one hand, both sexes increased their pornography consumption. On the other hand, girls with higher pornography consumption at baseline retained this pattern of consumption, whereas boys more frequently changed from lower pornography consumption to higher pornography consumption. Previous studies have reported that almost all boys consume pornography in their teens (6,10,16). It is commonly expected that boys watch pornography, and it can therefore be considered a normative behavior. It is less common for girls to consume pornography regularly (9,32), and it is unusual for girls to report higher pornography consumption.

There are concerns that pornography consumption affects public health (1,2). Individuals who watch sexually explicit media report feeling mentally diminished more frequently (1). This study partly confirms these findings. However, lower pornography consumption among male users was associated with depressive symptoms to a higher extent compared with higher pornography consumers. This may indicate that pornography consumption per se is an unproblematic and normalized behavior among boys and of limited threat to their psychological health and well-being. On the other hand, boys who do not watch pornography may differ from those who do and therefore may deserve special attention along with the boys who are frequent consumers because frequent users have reported more peer-relation problems than low and average consumers (6).

The causal effect of pornography consumption in relation to depressive symptoms is still unknown. It is likely that pornography consumption has different meanings for different individuals and is just one factor among many influencing young people’s mental health. It is important to understand that adolescents consume pornography for many reasons, and it is crucial for personnel who work with adolescents to acknowledge these differences and to treat adolescents individually rather than to assume that all adolescents are affected in the same way. Discussing pornography and the messages it conveys must be included in sexuality and relationship education in schools as well as in youth-friendly health-care settings involving adolescents. Few adolescents have discussed pornography with an adult (6), which means that a new generation is growing up with pornography as a main but undebated source of information about sex. It is of importance to continue efforts to evaluate how pornography consumption might affect psychosomatic and depressive symptoms over both shorter and longer periods of time. Another important aspect is that pornography consumption may have detrimental effects on people other than the consumer, for example creating a hostile environment at school (if consumed at school), sexual harassment, and sexual aggression behaviors. This implies that pornography consumption could affect peers indirectly and emphasizes the importance of including discussions about pornography in sexuality and relationship education.

This study has strengths and limitations. Both boys and girls participated, and the analyses were adjusted for sex, firstly, to avoid sex bias and, secondly, to investigate the interaction of sex, which enabled the conclusions based on sex differences. This study contributes information about the patterns of pornography consumption among girls, who have been underrepresented in previous studies. The sample was randomly selected from one medium-sized and one small city in one county in central Sweden. We believe that the results may be generalizable to Swedish adolescents in central Sweden since the chosen county is considered representative of Sweden, due to the proportion of inhabitants living in towns and rural areas, educational and income distribution, as well as the proportion of immigrants.

It is likely that the high number of students absent at follow-up affected the study results. A lower percentage of boys and participants born outside Sweden participated at follow-up. The increase of pornography consumption between baseline and follow-up could have been even larger, considering that boys and adolescent girls born outside Sweden were two groups consuming pornography consumption to a higher extent compared with peers. However, the dropout analysis showed few sociodemographic differences and no differences regarding psychosomatic and depressive symptoms between the dropouts and those who participated in both data collections. Nevertheless, the results should be interpreted with caution because just under half of the boys and just over half of the girls participated at both time points.

A possible weakness is that the participants’ responses may have been affected because the questionnaire was not anonymous. Even though a researcher visited each class and explained that the identification page would be separated from the questionnaire and available to the researchers only, some participants may still have felt discomfort about this. Questions about sexuality and pornography are very private, which may have caused some to be reluctant to participate even though it was emphasized that participation was voluntary. It was also possible to participate anonymously. Participants were asked about events that may have happened several years earlier, which may have caused recall bias. When using a self-reporting questionnaire, reliability should always be considered. However, the test–retest evaluation of the questionnaire in a similar study sample showed an acceptable degree of correlation between the two sets of results.

Furthermore, the scales used for depression and psychosomatic symptoms could be discussed. The Depression Self-Rating Scale (DSRS), in its adolescent version, has been frequently used in other adolescent populations (22,33,34) with results in line with other depression measures. Likewise, the composite scale of psychosomatic symptoms has been used in other Swedish adolescent populations (22,35,36). One could always argue that some of the six psychosomatic symptoms – headache, stomachache, nervousness, irritation, stress, and trouble sleeping – are more important than others. However, the symptoms are highly correlated, and since adolescence is a period of individual change we chose to use the composite measure, which we believe describes the psychosomatic symptom panorama (36) better than each symptom by itself. Moreover, the psychosomatic symptom scale was normally distributed, whereas the depressive symptom scale was positively skewed. We therefore used complementary statistics (Poisson regression) in our multivariable model of depression. The results from the Poisson regression showed two things: first, the findings were similar in both models, but with more significant main effects in in the Poisson model, and, second, the findings were not due to scaling artifacts.

The fact that data collection took place during different periods of the year (baseline, March–May; follow-up, January–March) could possibly contribute to seasonal effects on depressive symptoms. However, we do not think that this is a major bias since we measured the baseline depressive symptoms as an adjustment predictor in a model for depressive symptoms later in life. The problem would have been much more severe if the actual outcome, the follow-up data, had been measured during different periods in different subpopulations.

A final comment on the two outcome variables is the overlap between psychosomatic and depressive symptoms. Both sleeping problems and irritability in the index for psychosomatic symptoms are also criteria for depression, and the correlation between the scales was 0.605. However, we used both measurements as outcome variables in different models, so this intersection of symptoms is not a problem in the present analyses. Furthermore, it is possible that pornography consumption is related to common background variables, which in turn are associated to both psychosomatic and depressive symptoms. Such a common background variable could be social emotional regulation. This phenomenon is described as the outcome intersection problem, which is poorly described within the field of psychiatry (37).

Another weakness of the study was the measure of pornography consumption. First, it does not differentiate between intentional versus unintentional/accidental exposure. However, unintentional exposure turns into intentional in the same second as the individual continues to consume, if not forced to do so. Second, it does not examine whether exposure occurs when alone or as a part of a dyad/group. One might speculate that social activities could be protective. Pornography consumption in a group would therefore not necessarily threaten mental health unless involuntarily consumption (37). Third, the term pornography was not defined. In other words, the participants could define pornography as images of naked women/men or as images depicting individuals engaging in sexual activities. This could have led to certain categories of participants being more likely to embrace one definition. Associations to health outcomes could differ by different types of pornography, such as soft or violent pornography (4,39,40). We believe, however, that creating a more multi-dimensional measure of pornography, including the above-mentioned aspects, might generate more problems than it solves since such a variable by necessity has multiple dimensions, which all have to be investigated from different theoretical frameworks, as well as in different analytical models. Nonetheless, future studies should expand this topic by adjustment of several different aspects of pornography in analytical models. Another relevant next step would be to study if pornography consumption leads to sexual harassment behaviors as it has been established that sexual harassment victimization is associated with depressive symptoms. Pornography consumption may therefore be an indirect public health issue (41).

A common challenge in association research is the *cum hoc* fallacy, i.e. that two things that occur together must be causally related, as in this study, where there is an increase in pornography consumption as well as an increase in depressive and psychosomatic symptoms among adolescents (6,7,9,20,22). The present study has addressed this topic by using a longitudinal design as suggested in the literature (4,42). Another study design could have been a controlled experimental trial (42), which is both ethically and practically questionable, especially in an adolescent population.

## Conclusion

Higher pornography consumption at baseline predicted pornography consumption at follow-up as well as psychosomatic symptoms. By contrast, depressive symptoms were predicted by less pornography consumption at baseline. It appears that pornography consumption may, for some individuals, be associated to mental health issues. Differences between boys and girls and between adolescents with diverse ethnic backgrounds imply that counseling and discussion about pornography consumption need to be adjusted and individualized. Extensive pornography use is especially common among boys, which must be acknowledged among staff who meet adolescents in situations where sexuality and relationships are discussed, e.g. in youth centers and in youth-friendly school health services. For policy makers, it may be important to acknowledge that pornography consumption is part of many adolescents’ everyday life, therefore discussions related to pornography should be included in sexuality and relationship education in school. Further longitudinal studies are needed to track the development of mental health problems in relation to pornography consumption over time.

## Supplementary Material

Supplemental_online_material_Table_1.doc
