# Supplementary material for: Pornography consumption and psychosomatic and depressive symptoms among Swedish adolescents: a longitudinal study
Source: Ups J Med Sci. 2018 Nov 9;123(4):237–46. doi: 10.1080/03009734.2018.1534907 (PMC6327603; doi:10.1080/03009734.2018.1534907)
Supplement: Supplemental_online_material_Table_1.doc [file IUPS_A_1534907_SM2833.doc]

**Supplemental Online Material Table 1. Sociodemographic Backgrounds Among the Male and Female High School Students *(n* = 462).**

| Items | Total | Boys | Girls | *P*^a^ |
| --- | --- | --- | --- | --- |
|  | *n* = 462 (%) | *n* = 224 (%) | *n* = 238 (%) |  |
| Ethnic background   - Born in Sweden - Born outside Sweden | 431 (94)  28 (6) | 203 (91)  19 (9) | 228 (96)  9 (4) | 0.03 |
| Living with two parents | 303 (66) | 158 (71) | 145 (61) | ns |
| Living with one parent | 136 (30) | 58 (27) | 78 (35) | ns |
| Other | 22 (5) | 7 (3) | 15 (6) | ns |
| Dwelling   - Parent-owned - Rented | 347 (75)  115 (25) | 170 (76)  54 (24) | 177 (74)  61 (26) | ns |
| Father’s occupation   - Working/studying - Unemployed/on sick leave | 424 (92)  37 (8) | 208 (93)  15 (7) | 216 (91)  22 (9) | ns |
| Mother’s occupation   - Working/studying - Unemployed/on sick leave | 426 (92)  36 (7) | 204 (91)  20 (9) | 222 (93)  16 (7) | ns |
| Study program   - University preparation - Vocational | 269 (58)  193 (42) | 134 (60)  90 (40) | 135 (57)  103 (43) | ns |
| Pornography consumption   - Several times daily/ Daily - Weekly - A few times a month | 27 (9)  92 (29)  86 (27) | 27 (12)  87 (41)  72 (34) | 0 (0)  5 (5)  14 (13) | <0.001 |

^a^Chi-square tests, *P* < .05. ns: nonsignificant.
